# Single-cell Transcriptomic Analysis Reveals the Cellular Heterogeneity of Mesenchymal Stem Cells

**DOI:** 10.1016/j.gpb.2022.01.005

**Published:** 2022-02-03

**Authors:** Chen Zhang, Xueshuai Han, Jingkun Liu, Lei Chen, Ying Lei, Kunying Chen, Jia Si, Tian-yi Wang, Hui Zhou, Xiaoyun Zhao, Xiaohui Zhang, Yihua An, Yueying Li, Qian-Fei Wang

**Affiliations:** 1 CAS Key Laboratory of Genomic and Precision Medicine, Collaborative Innovation Center of Genetics and Development, Beijing Institute of Genomics, Chinese Academy of Sciences, Beijing 100101, China; 2 China National Center for Bioinformation, Beijing 100101, China; 3 University of Chinese Academy of Sciences, Beijing 100049, China; 4 Department of Medical Experimental Center, Qilu Hospital (Qingdao), Cheeloo College of Medicine, Shandong University, Qingdao 266035, China; 5 Qingdao Key Lab of Mitochondrial Medicine, Qingdao 266035, China; 6 International Department, Liangxiang Campus, Beijing University of Chinese Medicine, Beijing 102401, China; 7 Yihua Biotechnology Co., Ltd., Beijing 100041, China; 8 Peking University People’s Hospital, Peking University Institute of Hematology, Beijing 100044, China; 9 Department of Functional Neurosurgery, Third Medical Center, General Hospital of Chinese PLA, Beijing 100039, China

**Keywords:** Mesenchymal stem cell, Single-cell RNA sequencing, Heterogeneity, Lineage trajectory, Immune regulation

## Abstract

*Ex vivo*-expanded **mesenchymal stem cells** (MSCs) have been demonstrated to be a heterogeneous mixture of cells exhibiting varying proliferative, multipotential, and immunomodulatory capacities. However, the exact characteristics of MSCs remain largely unknown. By **single-cell RNA sequencing** of 61,296 MSCs derived from bone marrow and Wharton’s jelly, we revealed five distinct subpopulations. The developmental trajectory of these five MSC subpopulations was mapped, revealing a differentiation path from stem-like active proliferative cells (APCs) to multipotent progenitor cells, followed by branching into two paths: 1) unipotent preadipocytes or 2) bipotent prechondro-osteoblasts that were subsequently differentiated into unipotent prechondrocytes. The stem-like APCs, expressing the perivascular mesodermal progenitor markers *CSPG4/MCAM/NES*, uniquely exhibited strong proliferation and stemness signatures. Remarkably, the prechondrocyte subpopulation specifically expressed immunomodulatory genes and was able to suppress activated CD3^+^ T cell proliferation *in vitro*, supporting the role of this population in immunoregulation. In summary, our analysis mapped the heterogeneous subpopulations of MSCs and identified two subpopulations with potential functions in self-renewal and immunoregulation. Our findings advance the definition of MSCs by identifying the specific functions of their heterogeneous cellular composition, allowing for more specific and effective MSC application through the purification of their functional subpopulations.

## Introduction

Mesenchymal stem cells (MSCs) are multipotent cells that can be derived from various tissues, such as adult [adipose tissue, peripheral blood, and bone marrow (BM)] and neonatal [placenta, umbilical cord, and Wharton’s jelly (WJ)] tissues [1]. They possess self-renewal and multilineage differentiation capacities (such as osteocytic, adipocytic, and chondrocytic differentiation) [2], [3]. Furthermore, MSCs can secrete factors to regulate the inflammatory environment, support the development and maintenance of neurons, and promote the angiogenesis and wound healing [4], [5], [6]. Due to these properties, *ex vivo*-expanded MSCs have shown promise in cellular therapy and regenerative medicine applications in recent years.

MSCs exhibit two important cellular characteristics among their properties: a high proliferation ability with differentiation potential and an immunomodulatory capability. In culture, MSCs can be expanded to produce over 1 × 10^10^ cells from an initial population of 2–5 × 10^6^ cells over 30 days of culture [7]. Even after passaging up to 10 times, MSCs are still able to maintain their proliferative and multilineage differentiation capacities, two main characteristics that define the self-renewal ability of stem cells. However, the stem cell subsets responsible for these functions have yet to be identified [8]. Another important aspect of MSCs is their immunomodulatory plasticity via the release of soluble factors. In particular, their therapeutic immunosuppressive capacity is mainly achieved through the production of anti-inflammatory molecules, such as prostaglandin E2 (PGE2) and tumor necrosis factor-stimulated gene-6 (TSG-6), to inhibit the function of natural killer (NK) cells and effector T cells [4], [5], [6]. These findings suggest that MSCs may be a heterogeneous mixture of cells with diverse functions and multipotentiality. However, the potential cellular heterogeneity of MSCs still needs further characterization.

*In vitro* high-capacity assays have detected tripotent, bipotent, and unipotent clones [2], [3] derived from MSCs, indicating the significant heterogeneity of MSCs in clonogenicity and multilineage differentiation. Previous studies have applied single-cell RNA sequencing (scRNA-seq) to investigate the heterogeneity of *ex vivo* cultured human MSCs. However, limited cell subpopulations were identified. Huang et al. and Sun et al. have highlighted that one subpopulation has strong expression of genes involved in cell cycle progression, which prevents the inference of its potential cellular functions [9], [10]. And CD142^+^ WJ-derived MSCs (WJMSCs) have been identified with wound healing potential using bioinformatic analysis [10]. In addition, several studies only exhibited the differentially expressed genes (DEGs) and differential pathways after cell clustering but did not perform the functional assignment of the subpopulations [11], [12]. Other studies simply performed gene expression comparisons between MSCs by using scRNA-seq data from different sources, such as comparisons between WJ and BM, umbilical cord and synovial fluid, adipose and BM, and old and young BM [13], as well as from different stimulations, like interferon-gamma (IFN-γ) and tumor necrosis factor-a (TNF-a) [14]. Thus, the cellular heterogeneity of MSCs associated with the proliferation, multipotency, and immunomodulatory capabilities, as well as the differentiation trajectories, remains largely unclear. Biomarkers related to the enrichment of specific cells within the MSC population are also scarce.

To comprehensively investigate the cellular heterogeneity of MSCs, we profiled the single-cell transcriptomes of BM-derived MSCs (BMMSCs) and WJMSCs, two essential populations of MSCs from adult and neonatal tissues, respectively. Our data revealed that five MSC subpopulations with continuous developmental hierarchies existed among MSCs. We identified a stem-like active proliferative cell (APC) subpopulation, which exhibited a strong proliferation signature and high-level expression of the perivascular progenitor markers *CSPG4/MCAM/NES* as well as stemness signatures. The APC subpopulation was located at the apex of the differentiation trajectory. Following APCs on the trajectory was the lineage-primed multipotent mesenchymal progenitor cell (MPC) subpopulation, which exhibited features related to osteogenic, adipogenic, and chondrogenic lineages, simultaneously. Interestingly, a distinct prechondrocyte subpopulation highly expressed the genes encoding secreted immunomodulators and possessed greater potential to suppress activated CD3^+^ T cell proliferation, supporting the role of this subpopulation in immunoregulation. Overall, our study provides a single-cell transcriptomic blueprint of MSCs and uncovers the characteristics of stem-like, highly proliferative, multipotent, and immunoregulatory subpopulations among MSCs. These findings are helpful for advancing the definition of MSCs by identifying specific subpopulations, thereby enhancing their therapeutical potential by increasing specificity.

## Results

### Characteristics and single-cell transcriptomes of BMMSCs and WJMSCs

**Figure 1:**
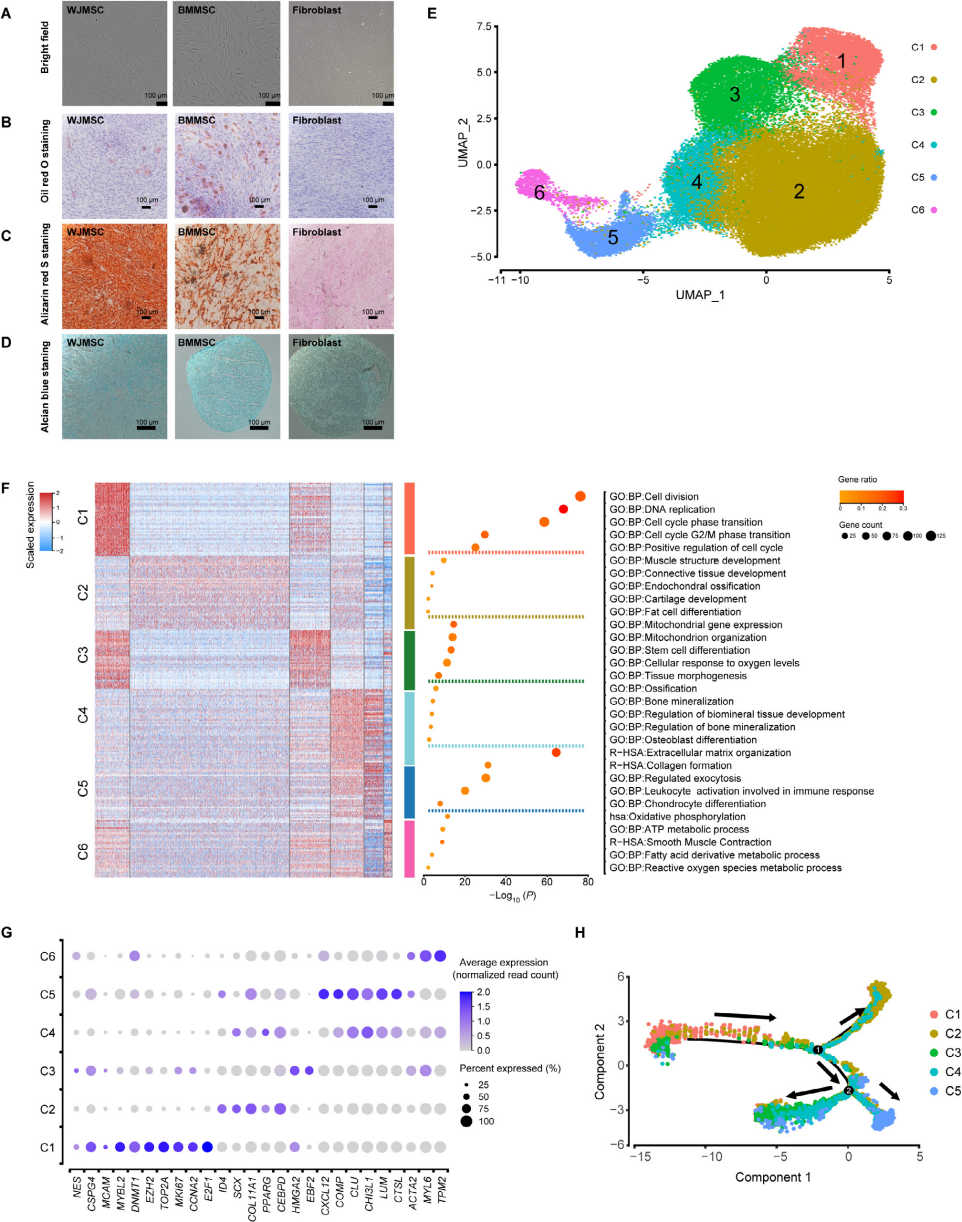
**Characteristics and single-cell transcriptome profiling of BMMSCs and WJMSCs** **A.** Representative bright-field images of the WJMSCs, BMMSCs, and fibroblasts (as a control). **B.** Representative images of WJMSCs/BMMSCs/fibroblasts stained with Oil Red O after adipogenic induction for 28 days. **C.** Representative images of WJMSCs/BMMSCs/fibroblasts stained with Alizarin Red S after osteogenic induction for 28 days. **D.** Representative images of WJMSCs/BMMSCs/fibroblasts stained with Alcian Blue after chondrogenic induction for 28 days. **E.** Cell type identification on the UMAP plot of 33,594 cells from 3 WJMSC samples and 27,702 cells from 3 BMMSC samples. **F.** DEGs and corresponding representative GO terms. Left: Heatmap showing the DEGs (expression percentage > 0.25, log_2_ FC > 0.4) in each cluster. The color indicates the scaled expression level. Right: Enriched functional pathways in each cluster are listed. The dot size indicates the number of genes, and its color indicates the gene ratio in each cluster (gene number *vs*. total gene number in the term). **G.** Dot plot showing the relative expression levels of the classical DEGs in each cluster. The dot size indicates the percentage of cells in the cluster expressing a gene; the shading indicates the relative level of expression (low to high shown as light to dark). **H.** Pseudotime map of each subpopulation generated by Monocle 2. MSC, mesenchymal stem cell; WJMSC, Wharton’s jelly-derived MSC; BMMSC, bone marrow-derived MSC; C1, Cluster 1; C2, Cluster 2; C3, Cluster 3; C4, Cluster 4; C5, Cluster 5; C6, Cluster 6; UMAP, uniform manifold approximation and projection; DEG, differentially expressed gene; GO, Gene Ontology; FC, fold change.

**Supplementary figure S1:**
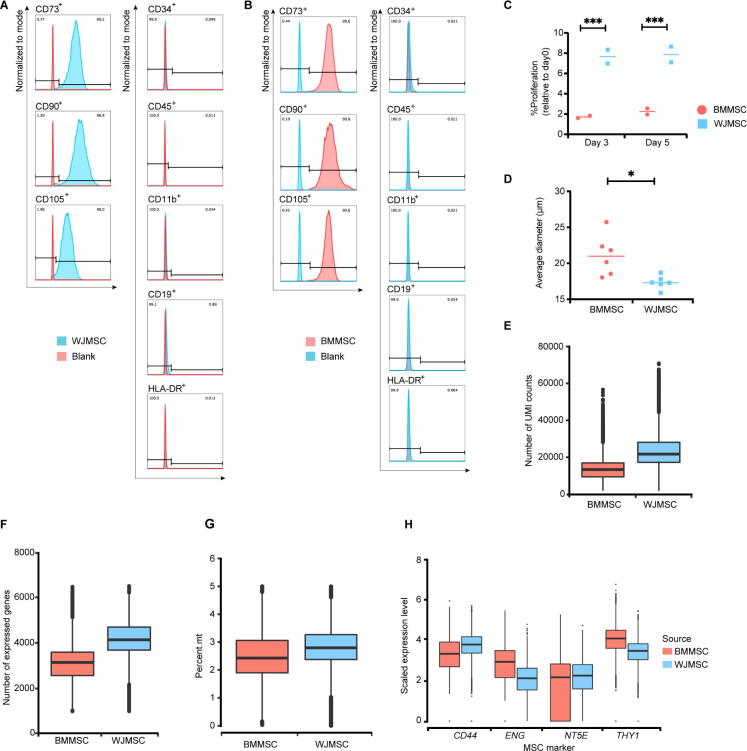
**Characteristics of MSCs and quality control of scRNA-seq data**. **A**. A representative flow diagram of the expression of positive markers (CD73, CD90, and CD105) and negative markers (CD34, CD45, CD11b, CD19, and HLA-DR) in WJMSCs. The proportion of cells with positive or negative expression is listed in the upper right or upper left corner. **B**. A representative flow diagram of the expression of positive markers (CD73, CD90 and CD105) and negative markers (CD34, CD45, CD11b, CD19, and HLA-DR) in BMMSCs. The proportion of cells with positive expression or negative expression is listed in the upper right or upper left corner. **C**. Histogram showing the proliferation ability as evaluated by the ratios of the cell counts on day 3 and day 5 relative to that on day 0. (*n* = 2, the data are expressed as the mean ± SD. ***, *P* < 0.005; analyzed by t-test.) **D**. Histogram showing the average diameters as analyzed with a Countstar instrument. (*n* = 6, the data are expressed as the mean ± SD. *, *P* < 0.05; analyzed by t-test.). **E.–G**. Box plot showing the number of UMI counts (E), expressed genes (F), and the percentage of mitochondrial genes (G) from single-cell transcriptomic data in WJMSCs and BMMSCs. **H**. Histogram showing the expression patterns of positive marker genes (CD73/*NT5E*, CD90/*THY1*, CD105/*ENG*, and *CD44*). The data are expressed as the mean ± SD. Percent.mt, the percentage of mitochondrial genes; UMI, unique molecular identifier; scRNA-seq, single cell RNA-sequencing.

BMMSCs and WJMSCs expanded *in vitro* at passages 6–7 were applied in our study. These cells were maintained in a stable state and were used for clinical application [15], [16], [17]. First, assays to identify and characterize MSCs were performed based on the criteria published by the Mesenchymal and Tissue Stem Cell Committee of the International Society for Cell & Gene Therapy (ISCT) [18]. The MSCs maintained their adherence to plastic when cultured under standard conditions and showed the common spindle-shaped, fibroblast-like morphology (Figure 1A). In an *in vitro* differentiation system, both BMMSCs and WJMSCs could differentiate into adipocytes (Figure 1B), osteoblasts (Figure 1C), and chondrocytes (Figure 1D). In addition, the proportions of MSCs expressing positive (CD73, CD90, and CD105) and negative (CD45, CD34, CD11b, CD19, and HLA‐DR) MSC markers were more than 98% and less than 1%, respectively (Figure S1A and B). However, in the same culture medium, WJMSCs had higher rates of proliferation (Figure S1C) and smaller average diameters than BMMSCs (Figure S1D), which is in line with the results of previous studies [1], [19], [20].

To uncover the cellular composition and diversity of MSCs, we performed scRNA-seq on 3 WJMSC and 3 BMMSC samples from different donors using the high-throughput 10x Genomics platform (Table S1). After stringent cell filtration, high-quality single-cell transcriptomes of 61,296 MSCs (33,594 WJMSCs, accounting for 54.8% of the total population; 27,702 BMMSCs, accounting for 45.2% of the total population) were obtained for downstream analysis. Compared to BMMSCs, WJMSCs had a higher median number of expressed genes (4136 for WJMSCs *vs* . 3144 for BMMSCs) and higher unique molecular identifier (UMI) counts (21,730 for WJMSCs *vs.* 13,317 for BMMSCs) (Figure S1E and F) but a similar median percentage of mitochondrial genes (2.79% for WJMSCs *vs*. 2.43% for BMMSCs) (Figure S1G). The average expression levels of the well-known MSC markers *CD73/NT5E*, *CD90/THY1*, *CD105/ENG*, and *CD44* (Figure S1H) were consistently high in both WJMSCs and BMMSCs. These results indicated that we successfully obtained single-cell transcriptomes of WJMSCs and BMMSCs via a high-throughput approach for further analysis.

### Transcriptional heterogeneity exists within five distinct MSC subpopulations with unique signatures

**Supplementary figure S2:**
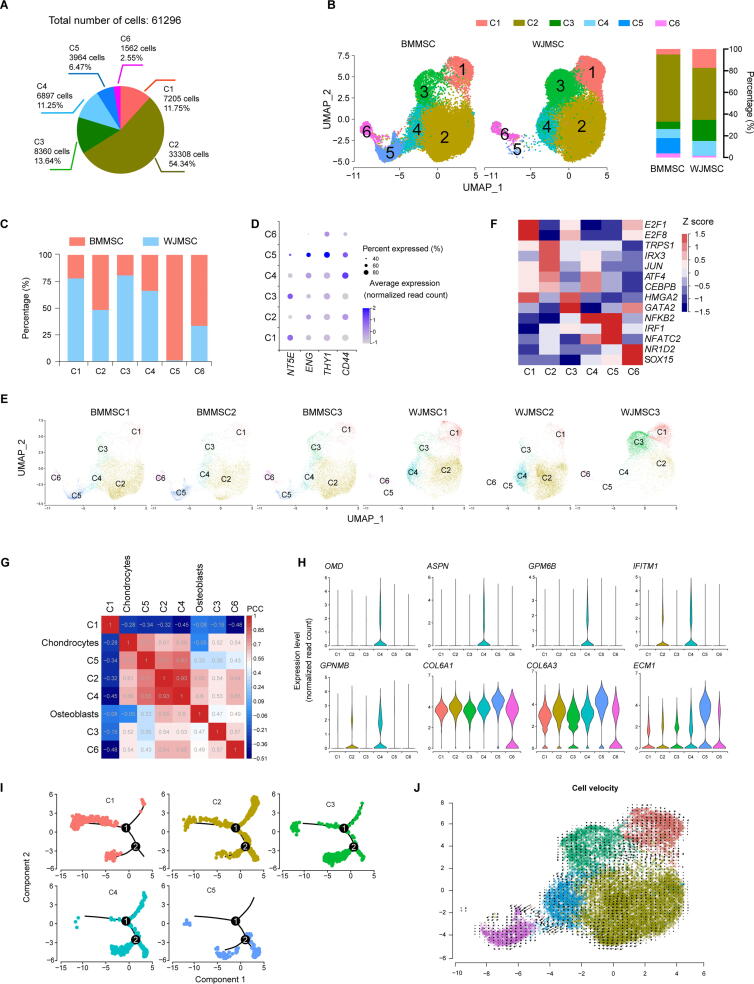
**Cellular taxonomy of MSCs by scRNA-seq**. **A**. Pie chart showing the cell number and proportion of each subpopulation. **B**. Sample distribution. Left: UMAP plot showing the cell distribution of the two MSC sources (WJMSCs and BMMSCs). Right: Histogram showing the proportion of each subpopulation from the two MSC sources (WJMSCs and BMMSCs). **C**. Histogram showing the percentage of the two MSC sources in each subpopulation. **D**. Dot plot showing the relative expression levels of positive marker genes (CD73/*NT5E*, CD90/*THY1*, CD105/*ENG*, and *CD44*). The dot size indicates the proportion of cells in the cluster expressing a gene; the shading indicates the relative level of expression (low to high, shown as light to dark). **E**. UMAP plot showing the cell distribution of the six individual samples. **F**. Heatmap showing gene name encoding activated TFs predicted by SCENIC. G. Heatmap of the correlation matrix of transcriptome between our MSC subpopulations and specific cells from published dataset [34], including osteoblasts and chondrocytes. H. Violin plots showing the expression levels of selected genes (*OMD, ASPN, GPM6B, IFITM1, GPNMB, COL6A3, COL6A1*, and *ECM1*) in each cluster. I. Monocle2 plot colored by each cell cluster identity. J. Velocity field projected onto the UMAP embedding. Arrows show the local average velocity evaluated on a regular grid. TFs, transcription factors.

To investigate the cellular heterogeneity of MSCs, unsupervised clustering by the uniform manifold approximation and projection (UMAP) technique was performed after cell cycle regression. In total, six clusters were identified (Figure 1E, Figure S2A–C; Table S2). Typical MSC markers, including *CD73/NT5E*, *CD90/THY1*, *CD105/ENG*, and *CD44*, showed variable expression levels among each cluster (Figure S2D), suggesting that the traditional criteria were unable to define MSC subpopulations due to their intrinsic heterogeneity. Meanwhile, based on the UMAP patterns of individual samples from these six donors, we found that BMMSCs had a more similar pattern while WJMSCs had higher individual complexity, suggesting that WJMSCs possessed higher inter-donor variability (Figure S2E). To determine the cellular identity of each cluster, DEGs and corresponding enriched pathways, as well as potential key regulators, were identified. Cells in Cluster 1 showed a stronger characteristic of active proliferation, as these cells had high-level expression of genes related to DNA replication and cell cycle progression, including proliferation markers (*TOP2A*, *MKI67*, and *E2F1*) and a cell cycle regulator (*CCNA2*) (Figure 1F and G). Furthermore, the transcription factor (TF) genes *E2F1* and *E2F8*, known cell cycle progression regulators, were predicted by Single Cell Regulatory Network Inference and Clustering (SCENIC v1.1.2-2) to be the active TFs in Cluster 1 (Figure S2F). Interestingly, *NG2/CSPG4*, *CD146/MCAM*, and *NES* (Figure 1G), the characteristic markers of perivascular mesodermal progenitor cells [21], [22], [23], were also highly expressed in cells in Cluster 1. When these observations were combined with the findings regarding the expression levels of genes essential for maintaining pluripotency and the undifferentiated stem cell state, such as *MYBL2* [24], *DNMT1*, and *EZH2* [25] (Figure 1G), Cluster 1 cells were classified as potentially stem-like APCs.

Cells in Cluster 2, accounting for more than half of the total cell number (Figure S2A), exhibited an expression signature enriched for trilineage differentiation, including osteogenic differentiation (*ID4* [26]), chondrogenic differentiation (*SCX* [27] and *COL11A1* [28]), and adipogenic differentiation (*PPARG* and *CEBPD*) [29] (Figure 1F and G). The predicted TF genes in Cluster 2 are known to govern diverse lineage commitment decisions, including mesoderm development (*IRX3*), osteogenesis (*JUN* and *ATF4*) [30], chondrogenesis (*TRPS1*) [31], and adipogenesis (*CEBPB*) [29] (Figure S2F). Considering the revealed multilineage differentiation potential, cells in Cluster 2 were referred to as tripotent multipotent MPCs.

In addition to stem and progenitor cells, differentiated precursors were identified. Cells in Cluster 3 were enriched with genes involved in stem cell differentiation (*PSMD2*, *PSMD7*, and *PHF5A*), tissue morphogenesis (*TBX3*, *CFL1*, and *TRIM28*), and mitochondrial biogenesis for adipogenesis (*NDUFA9*, *UQCC2*, *ATP5F1B*, and *COX20*) (Figure 1F). Moreover, Cluster 3 cells expressed high levels of genes related to the regulation of adipocyte differentiation, such as *EBF2* and *HMGA2* (Figure 1G). *GATA2*, reported to be expressed in preadipocytes and play a central role in controlling adipogenesis [32], [33], was also predicted to be the active TF gene in Cluster 3 (Figure S2E). Thus, Cluster 3 cells were referred to as unipotent preadipocytes.

Cells in Cluster 4 expressed high levels of the cartilage-specific gene *COMP* and the extracellular matrix remodeling genes *CHI3L1*, *CLU*, *LUM*, and *CTSL* (Figure 1G). Correlation analyses of the top-gene transcriptome between our analyzed MSCs and published chondrocyte and osteoblast datasets [34] revealed that cells in Cluster 4, which also showed higher expression of osteogenesis-related genes (*OMD*, *ASPN*, *GPM6B*, *IFITM1*, and *GPNMB*) (Figure S2G and H), were closely related to chondrocytes and osteoblasts. Thus, cells in Cluster 4 were referred to as bipotent prechondro-osteoblasts (pre-COs). Cluster 5, on the other hand, resembled chondrocytes and had higher expression of genes involved in chondrogenesis (*COL6A3*, *COL6A1*, and *ECM1*) (Figure S2G and H). Interestingly, pathways involved in immunomodulation and secretion were also enriched in Cluster 5 (Figure 1F). Thus, Cluster 5 cells were annotated as immunoregulatory prechondrocytes. Cells in Cluster 6, accounting for the lowest proportion (2.25%) of the cell population (Figure S2A), were enriched with genes essential for smooth muscle contraction (*ACTA2*, *MYL6*, and *TPM2*) (Figure 1F and G). The predicted active regulatory TF genes *SOX15* and *NR1D2* have been demonstrated to play a key role in determining early myogenic cell development (Figure S2E) [35], [36]. Thus, Cluster 6 cells were referred to as pre-smooth muscle cells (pre-SMCs), consistent with the capability of MSCs to differentiate toward vascular lineages. Cells in Cluster 6 also expressed high levels of genes participating in metabolic processes (Figure 1F), supporting the importance of metabolic reprogramming during the differentiation of MSCs into SMCs [37].

To explore the relations and the developmental hierarchies among the subpopulations, we performed pseudotime analysis with Monocle2. The stem-like APCs were positioned in the “source” cell state, followed by MPCs. Then, two branching paths were derived from MPCs: one leading to pre-COs and differentiated unipotent prechondrocytes, and the other leading to preadipocytes (Figure 1H, Figure S2I). Moreover, the Monocle2 result was supported by the RNA velocity analysis with Velocyto [38] (Figure S2J), which enables the prediction of potential directional trajectories and cell state transitions by connecting measurements to the underlying mRNA splicing kinetics. Overall, these findings reveal that MSCs are composed of heterogeneous and continually developing cell populations that progress from stem-like APCs to tripotent MPCs and ultimately to bipotent and unipotent precursors.

### Specialized APCs (Cluster 1) possess stem-like transcriptional signatures

It is generally believed that one of the key characteristics of MSCs is their ability to undergo robust proliferation while maintaining their multilineage differentiation potential. We performed further analysis to explore whether Cluster 1 cells possess this stem-like characteristic.

**Figure 2:**
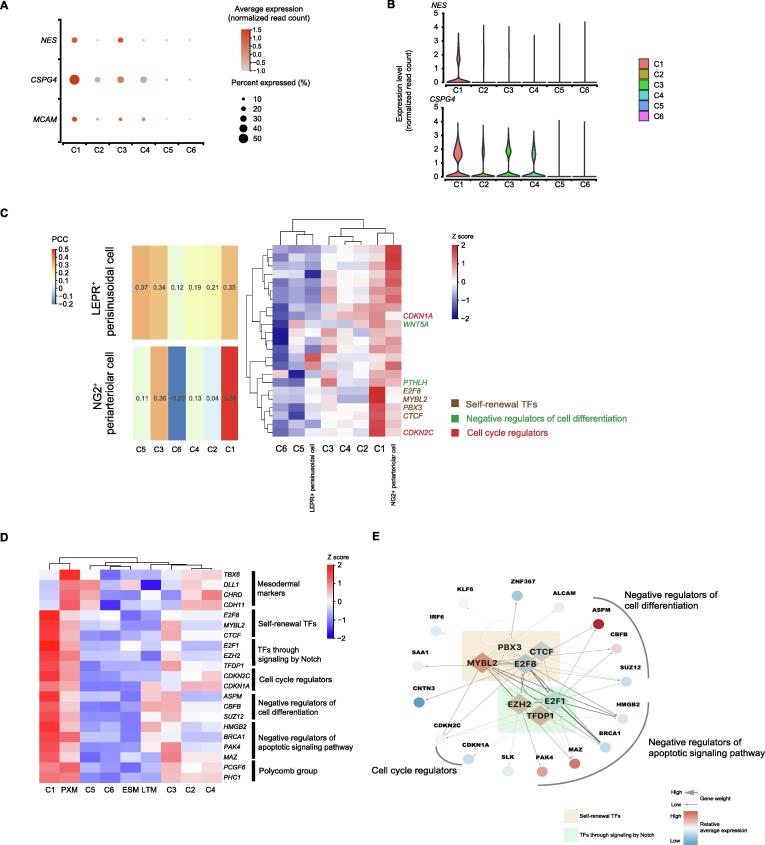
**Identification of specialized stem-like cells (Cluster 1) with high proliferative capacity** **A.** Dot plot showing the relative expression levels of mesodermal progenitor cell marker genes (*NG2/CSPG4*, *CD146/MCAM*, and *NES*) in MSC subpopulations. The dot size indicates the percentage of cells in the cluster expressing a gene; the shading indicates the relative level of expression (low to high shown as light to dark). **B.** Violin plot showing the relative expression levels of the mesodermal progenitor cell marker genes (*NG2/CSPG4* and *NES*) in MSC subpopulations. **C.** Comparison between single-cell transcriptomes of MSC subpopulations and cells from published datasets. Left: Heatmap displaying the correlation matrix analyzed by PCC. Right: Expression patterns of selected genes involved in the indicated biological processes between the transcriptomes of MSC subpopulations and cells from published datasets [42], including NG2^+^ periarteriolar cells and LEPR^+^ perisinusoidal cells. **D.** Heatmap displaying the expression patterns of selected genes involved in the indicated biological processes between each MSC subpopulation and cells from published datasets [43], including DLL1^+^ PXM, LTM, and ESM. **E.** Schematic illustrating the regulation of DEGs by potential activated TFs in Cluster 1. The potential activated TFs for Cluster 1 predicted by SCENIC are shown as diamonds, and their potential downstream targets are shown as circles. PXM, paraxial mesoderm; LTM, lateral mesoderm; ESM, early somite; PCC, pearson correlation coefficient; TF, transcription factor.

**Supplementary figure S3:**
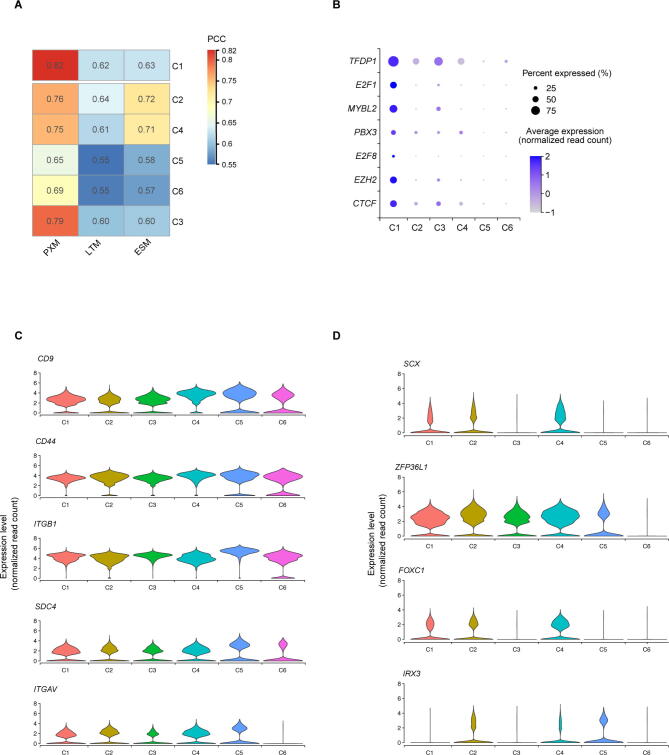
**Cluster 1 cells exhibited a stem-like transcriptional signature**. **A**. Heatmap of the correlation matrix between the transcriptome of each subpopulation and cells from published datasets [43], including DLL1+ PXM, LTM, and ESM. **B**. Dot plot showing the relative expression levels of the activated transcription factors (*CTCF, EZH2, E2F8, PBX3, MYBL2, E2F1*, and *TFDP1*) in Cluster 1 predicted by SCENIC. **C**. Violin plots showing the expression levels of stemness-associated markers (*CD9, CD44, ITGB1, SDC4*, and *ITGAV*) in each cluster. **D**. Violin plots showing the expression levels of genes involved in mesodermal development (*SCX, ZFP36L1, FOXC1*, and *IRX3*) in each cluster.

Compared with cells in the other clusters, Cluster 1 cells expressed extremely high levels of *NG2/CSPG4*, *CD146/MCAM*, and *NES* (Figure 2A and B). Previously, NESTIN^bright^ NG2/CSPG4^+^ periarteriolar mesodermal progenitor cells, whose marker phenotype indicates the stemness characteristics of self-renewal and differentiation into multiple mesenchymal lineages, have been reported to differentiate into MSCs and to constitute the origin of MSCs in multiple organs [21], [23]. In addition, the paraxial mesoderm (PXM) can bud off into MSCs in both *in vitro* and *in vivo* experiments [39], [40], [41]. To validate the identity of Cluster 1, we compared the single-cell transcriptome data between Cluster 1 cells and NG2^+^ periarteriolar cells, LEPR^+^ perisinusoidal cells [42], and PXM cells, respectively [43]. We compared the overall expression pattern by performing a Pearson correlation test with genes involved in maintaining stemness, including those related to self-renewal (*E2F8*, *CTCF*, *PBX3*, and *MYBL2*), negative regulation of cell differentiation (*ASPM*, *CBFB*, *SUZ12*, *WNT5A*, and *PTHLH*), and cell cycle regulation (*CDKN2C* and *CDKN1A*). Clearly, cells in Cluster 1 but not cells in other clusters were closely related to NG2^+^ periarteriolar cells (*in vivo*;Figure 2C) and PXM cells (*in vitro*; Figure 2D, Figure S3A). The stemness genes and those related to notch signaling pathways (*E2F1*, *EZH2*, and *TFDP1*), negative regulation of apoptosis (*HMGB2*, *BRCA1*, *PAK4*, and *MAZ*), and polycomb groups (*PCGF6* and *PHC1*) were all strongly co-expressed in Cluster 1 (Figure 2C and D). These observations indicated that Cluster 1 cells resembled mesodermal progenitor cells with the ability to self-renew and differentiate into multiple mesoderm lineages. Via SCENIC analysis, *CTCF*, *EZH2*, *E2F8*, *PBX3*, *MYBL2*, and *TFDP1* were also identified as the genes encoding potential activated TFs in Cluster 1. These TFs are transcriptional activators targeting the genes related to self-renewal pathways (Figures 2E, Figure S3B). Together, these results suggest that Cluster 1 cells possess a high proliferative capacity combined with stem-like transcriptional signatures.

### MPCs (Cluster 2) are subgrouped into trilineage orientations

**Figure 3:**
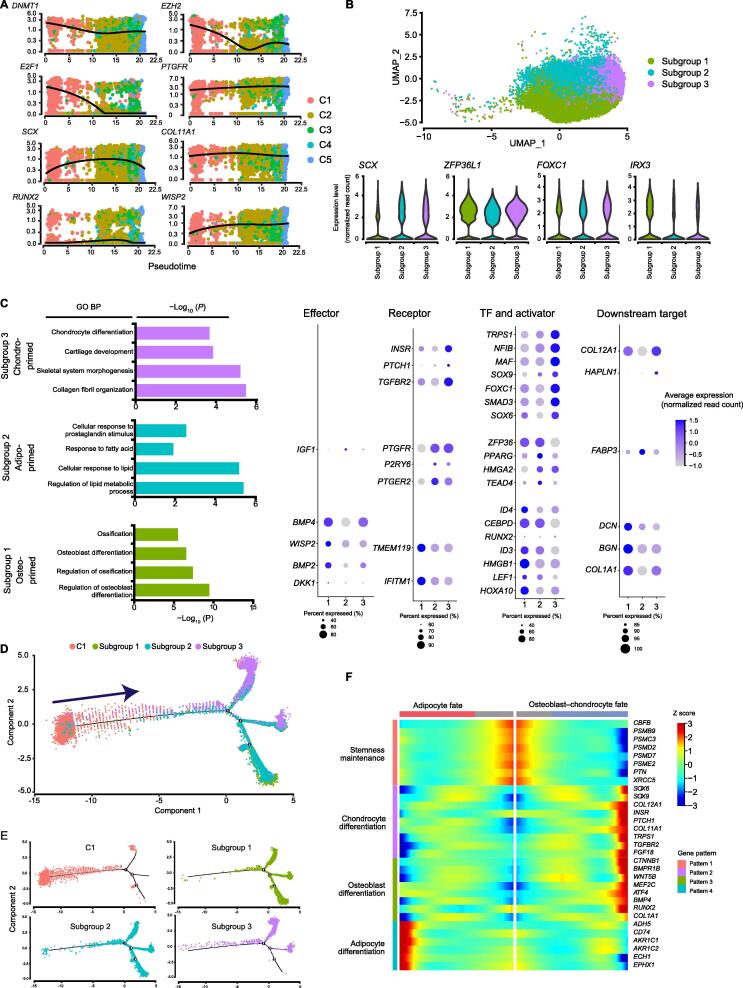
**Cluster 2 MPCs are primed towards trilineage orientations** **A.** Relative expression patterns across pseudotime of representative genes for self-renewal maintenance (*DNMT1*, *EZH2*, and *E2F1*), adipogenesis (*PTGFR*), chondrogenesis (*SCX* and *COL11A1*), and osteogenesis (*RUNX2* and *WISP2*). The dots are colored by cluster name. **B.** Cell type identification on the UMAP plot of MPCs (*n* = 30,946 cells) (top) and Violin plots showing the relative expression levels of genes involved in mesodermal development (*ZFP36L1*, *FOXC1*, *IRX3*, and *SCX*) in each subgroup of MPCs (bottom). **C.** Representative GO terms and corresponding DEGs. Left: Representative GO BP terms enriched with up-regulated genes (average log_2_ FC > 0.25) in each subgroup of MPCs. Right: Dot plot showing the relative expression levels of representative genes involved in the indicated terms. The dot size indicates the percentage of cells in the cluster expressing a gene; the shading indicates the relative level of expression (low to high shown as light to dark). **D.** Developmental trajectory between Cluster 1 and each subgroup from MPCs. The pseudotime map of Cluster 1 and each subgroup from MPCs was generated by Monocle2. **E.** Monocle2 plot colorred by each cell cluster identity. **F.** BEAM analysis by Monocle2 showing the different expression patterns during the development of stem-like MSCs to an osteoblast–chondrocyte or adipocyte fate. MPC, mesenchymal progenitor cell; BP, biological process; BEAM, branched expression analysis modeling.

Cells in Cluster 2, annotated as multipotent MPCs, expressed MSC stemness-associated markers (*CD9*, *CD44*, *ITGB1*, *SDC4*, and *ITGAV*) (Figure S3C). Moreover, the dot plot (Figure 1F) and pseudotime model of gene expression dynamics (Figure 3A) both reflected that the MPCs co-expressed osteogenesis-, chondrogenesis- and adipogenesis-associated genes involved in early-stage transcriptional programs at an intermediate level compared with that in the primitive-state (Cluster 1) and commitment-state (Clusters 3–5) cells. Transcripts in the osteogenesis program included the upstream transcriptional regulator gene *ID4*. Transcripts in the chondrogenesis program included the key TF gene *SCX* and the downstream target extracellular matrix protein gene *COL11A1*. Transcripts in the adipogenesis program included the key TF gene *PPARG* and the upstream transactivator gene *CEBPD* (Figure 1G). In addition, the predicted TFs in MPCs were related to driving diverse lineage commitment decisions (Figure S2F). This model of MPCs is similar to a model proposed in a previous study, which suggests that multipotent progenitor cells can exist in a lineage-priming state and that lineage-affiliated genes are “primed” for expression later during differentiation [44]. Thus, our results support the concept that lineage priming might occur in MPCs.

Although MPCs are multipotent and possess trilineage differentiation potential, whether these abilities are executed by a single cell population or distinct subgroups of cells is unclear. To explore the diverse progenitors for specific lineages, we subclustered the MPCs by unsupervised clustering via UMAP (Figure 3B). Interestingly, except for the uniform expression of genes involved in mesodermal development (*ZFP36L1*, *FOXC1*, *IRX3*, and *SCX*) (Figures 3B, Figure S3D), there were three subgroups showing distinct expression patterns. The controllers of transcriptional programs related to osteogenesis, chondrogenesis, and adipogenesis, such as the TF genes *RUNX2, SCX*, and *PPARG*, respectively, were expressed at relatively high levels in the aforementioned subgroups. In addition, cells in subgroup 1 expressed genes related to osteoblast differentiation (*IFITM1* and *TMEM119*) and osteoblast progenitor cell proliferation (*COL1A1*, *ID3*, and *ID4*) (Figure 3C). Thus, cells in subgroup 1 were referred to as osteo-primed MPCs. Cells in subgroup 2 expressing genes related to fatty acid metabolism and lipid accumulation (*IGF1*, *PPARG*, *FABP3*, *P2RY6*, *PTGER2*, and *PTGFR*) (Figure 3C) were referred to as adipo-primed MPCs. Cells in subgroup 3 expressed key genes involved in aspects of chondrogenesis, including chondrocyte differentiation, cartilage development, and collagen fibril organization (*COL11A1*, *COL12A1*, *MAF*, *NFIB*, *TGFBR2*, *TRPS1*, *FGF18*, and *INSR*). Thus, subgroup 3 cells were referred to as chondro-primed MPCs. Therefore, three subgroups of MPCs with differentiation bias, with the possibility of leading to distinct differentiation programs, were identified.

To elucidate the early cell development program of MSCs, we performed developmental trajectory analysis with stem-like APCs (Cluster 1), osteo-primed MPCs, adipo-primed MPCs, and chondro-primed MPCs (Figure 3D). Two major routes of differentiation from the initial stem-like APCs to the three subgroups of MPCs were revealed, and each route was associated with more than one subgroup of MPCs (Figure 3E). We hypothesized that MPCs in lineage-priming states may adopt stochastic and reversible fates rather than stable states. Then, to investigate the different regulatory patterns of gene expression during this early transition, we performed branched expression analysis modeling (BEAM) on the first bifurcation point with Monocle2. Hierarchical clustering was performed with the DEGs during specification, resulting in the identification of four different gene expression patterns during trilineage development (Figure 3F). The genes enriched in pattern 1 were related to stemness maintenance and were highly expressed in prebranched APCs. These genes included stemness-associated molecules (*CBFB* and *PTN*) and genes encoding the proteasome complex subunits (*PSMB9*, *PSMC3*, *PSMD2*, *PSMD7*, and *PSME2*), which play pivotal roles in the regulation of self-renewal, pluripotency, and differentiation of stem cells [45], [46] (Figure 3F). Patterns 2 and 3, containing the genes that were up-regulated in osteo–chondro-committed precursors, were enriched with chondro-specific genes such as *SOX6*, *SOX9*, and *COL12A1* [47], as well as the osteo-specific genes *ATF4* and *RUNX2* (Figure 3F) [30], [48], [49]. Pattern 4 contained the genes that were up-regulated in adipo-committed precursors (Figure 3F), such as genes encoding aldo–keto reductases (*AKR1C1* and *AKR1C2*) and *EPHX1*, which are vital for adipocyte differentiation [50], [51]. These results are consistent with the balance toward adipogenesis in favor of osteo–chondrogenesis during MSC commitment [47], [52]. Considering these results collectively, we found that MPCs reasonably exist as a transition state between declining stem cell activity and ongoing progression to osteoblast/chondrocyte/adipocyte fate.

### Prechondrocytes (Cluster 5) specifically harbor immunoregulatory capacity

**Figure 4:**
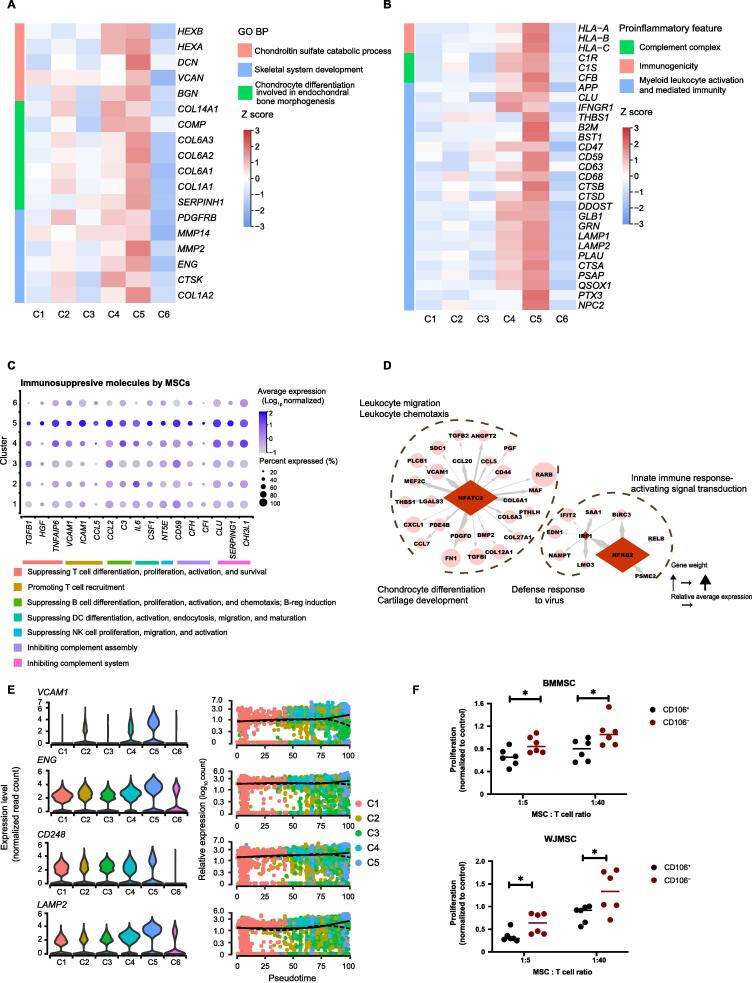
**BMMSC-dominant prechondrocytes (Cluster 5) specifically harbor immunoregulatory capacity** **A.** Heatmap showing the relative expression levels of selected genes involved in chondrogenesis in each subpopulation of MSCs. The color indicates the scaled expression level. **B.** Heatmap showing the relative expression levels of selected proinflammatory genes in each subpopulation. The color indicates the scaled expression level. **C.** Dot plot showing the relative expression levels of selected genes involved in immunosuppression in each subpopulation. The dot size indicates the percentage of cells in the cluster expressing a gene; the shading indicates the relative level of expression (low to high shown as light to dark). **D.** Schematic illustrating the regulation of DEGs by potential activated TFs in Cluster 5. The potential activated TFs in Cluster 5 predicted by SCENIC are shown as diamonds, and their potential downstream targets are shown as circles. **E.** Violin plots (left) and pseudotime trajectories (right) showing the relative expression levels of representative potential markers. **F.** Proliferation of activated CD3^+^ T cells (stimulated with anti-CD2/CD3/CD28 coated microbeads) when cocultured with CD106^+^ or CD106^−^ MSCs at 1:5 or 1:40. “Proliferation” was measured as the percentage of FSC^high^Dye^low^ cells in the living cells, and was normalized with respect to the percentage of activated CD3^+^ T cells without coculture with MSCs. Data are represented as mean ± SD (*n* = 6). *, *P* < 0.05 (*t*-test).

**Supplementary figure S4:**
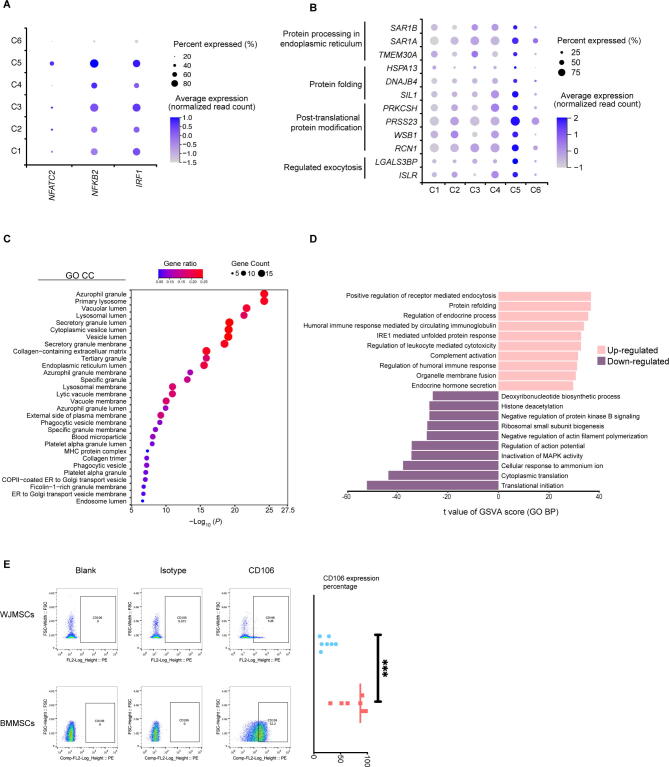
**Cluster 5 cells exhibited an immunoplasticity transcriptional signature**. **A**. Dot plot showing the relative expression levels of predicted TFs in Cluster 5 (*NFATC2, NFKB2*, and *IRF1*). The dot size indicates the proportion of cells in the cluster expressing a gene; the shading indicates the relative level of expression (low to high, shown as light to dark). **B**. Dot plot showing the relative expression levels of selected genes involved in the indicated biological processes in Cluster 5. The dot size indicates the proportion of cells in the cluster expressing a gene; the shading indicates the relative level of expression (low to high, shown as light to dark). **C**. GO analysis with g:Profiler showing CC terms enriched with differentially expressed genes in Cluster 5. The gene count is indicated by the dot size, and the gene ratio is indicated by the color of the dot (blue, low ratio; red, high ratio). **D**. Histogram showing representative enriched GO terms involved in protein processing in Cluster 5 identified by GSVA. **E**. Representative flow cytometry plots and statistical data of CD106/*VCAM1* expression levels in MSCs from WJMSCs and BMMSCs (n = 7, the data are expressed as the mean ± SD. ***, *P* < 0.005; analyzed by t-test.). CC, cellular component; GSVA, gene set variation analysis.

Genes involved in the chondrogenesis process, including the chondroitin sulfate catabolic process, endochondral bone morphogenesis, and skeletal system development, were highly expressed in Cluster 5 (Figure 4A). In addition, Cluster 5 enriched functional processes involved in the presentation of proinflammatory features, including immunogenicity, complement system activation or inhibition, and myeloid leukocyte activation, as well as anti-inflammatory features, such as suppression of the proliferation, differentiation, and activation of immune cells (*e.g.*, T cells, B cells, NK cells, and dendritic cells) (Figure 4B and C). The predicted TF genes, such as *IRF1*, *NFATC2*, and *NFKB2*, and their enriched co-expressed gene sets (Figure 4D, Figure S4A), are important regulators of the innate and acquired immune responses [53], [54]. These results suggested that Cluster 5 cells, referred to as prechondrocytes, possessed immunoregulatory potential.

In addition to their immunomodulatory profile, Cluster 5 cells expressed high levels of genes related to protein processing in the endoplasmic reticulum (*SAR1A*, *SAR1B*, and *TMEM30A*), protein folding (*HSPA13*, *DNAJB4*, and *SIL1*), posttranslational protein modification (*PRKCSH*, *PRSS23*, *WSB1*, and *RCN1*), and regulation of exocytosis (*LGALS3BP* and *ISLR*) (Figure S4B), consistent with the results of previous studies suggesting that factors in the MSC secretome might perform the major tasks in MSC-mediated immunoregulation [4]. To further clarify how Cluster 5 cells regulate inflammation and immune responses, cellular component enrichment analysis was performed using g:Profiler [55]. The results showed that most immunomodulatory genes were localized in the extracellular space or extracellular vesicles (Figure S4C). Evaluation of known pathway expression patterns in Cluster 5 via gene set variation analysis (GSVA) revealed strong enrichment of pathways such as positive regulation of receptor-mediated endocytosis, regulation of endocrine process, organelle membrane fusion, and endocrine hormone secretion (Figure S4D). Together, these results suggested that the immunoregulatory effect of Cluster 5 cells is likely due to their production of exosomes or secretion of soluble factors.

**Supplementary figure S5:**
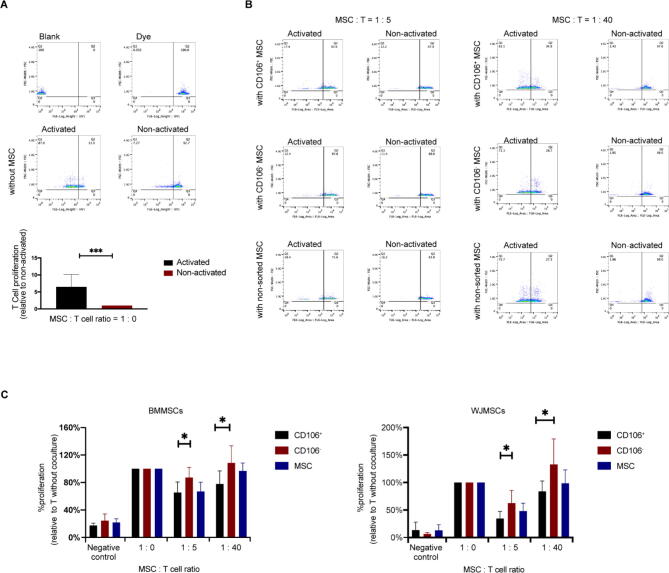
**The immunoregulatory capacity of Cluster 5 was supported by the stronger inhibition on activated CD3+ T cells**. **A**. A representative flow diagram (up) and histogram (bottom) showing T cell proliferation with and without CD2/CD3/CD28 stimulation (n = 8, the data are expressed as the mean ± SD. ***, *P* < 0.005; analyzed by t-test; normalized with respect to the percentage of non-activated T cells). **B**. A representative flow diagram of T cell proliferation co-cultured with CD106+/CD106-/non-sorted MSCs with and without activation. **C**. Histogram depicting T cells proliferation stimulated with anti-CD2/CD3/CD28 coated microbeads co-culturing with CD106+/CD106-/non-sorted MSCs at 1:5 or 1:40, the data were analyzed by dye loss and FSC/SSC gating, respectively. The data were relative to the positive control group, that was the percentage of activated T cells without coculture with MSCs (n = 6, the data are expressed as the mean ± SD. ***, *P* < 0.005; analyzed by one-way ANOVA followed by Tukey's post-hoc test). FSC, forward scatter; SSC, side scatter.

MSCs can modulate the response of immune cells via interaction with lymphocytes, especially T cells, via both the innate and adaptive immune systems to produce anti-inflammatory effects after homing to sites of inflammation *in vivo*. To test the ability of Cluster 5 cells to counteract inflammation, we purified Cluster 5 cells by the surface marker CD106, which was identified as the most significant and specific marker in our data (Figure 4E, Figure S4E). When cocultured with activated CD3^+^ T cells, both CD106^+^ WJMSCs and BMMSCs reduced CD3^+^ T cell proliferation more significantly than their corresponding CD106^−^ cells (Figure 4F, Figure S5A–C), indicating that CD106^+^ Cluster 5 MSCs exhibited greater anti-inflammatory capacity than other MSC subpopulations. Taken together, these observations suggest that Cluster 5 MSCs, identified as prechondrocytes, possess both proinflammatory and anti-inflammatory potentials mediated in a paracrine manner by a variety of secreted factors.

### Cultured MSCs show different properties from primary MSCs by single-cell analysis

**Supplementary figure S6:**
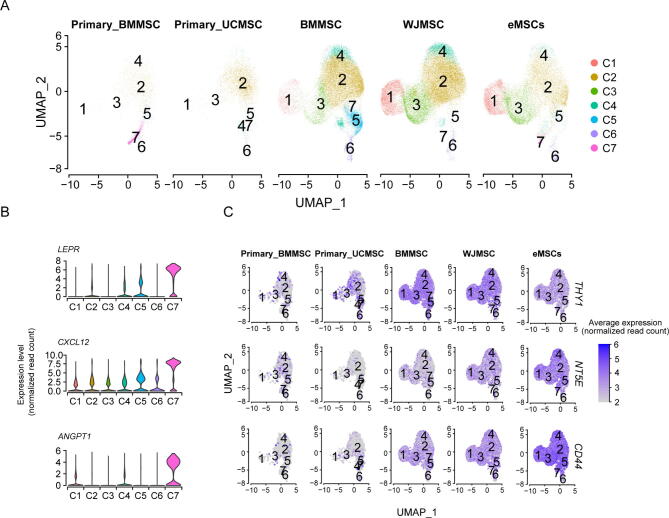
**The integration analysis between our dataset and published dataset**. **A**. UMAP plot showing the source distribution from the integration analysis, including the BMMSCs and WJMSCs from our data and the cultured human endometrial MSCs [57] , uncultured human UCMSCs [12], and primary BMMSC [56] from published scRNA-seq data. **B**. Violin plots showing the expression levels of genes involved in mesodermal development (*LEPR*, *CXCL12*, and *ANGPT1*) in each cluster. **C**. The feature plot shows the expression of MSC markers (CD73/*NT5E*, CD90/*THY1*, and *CD44*) in each source. UCMSCs, umbilical cord MSCs; eMSCs, endometrial MSCs.

To explore the similarity and difference between cultured MSCs and uncultured primary MSCs, we further performed integrated analyses of our data with published scRNA-seq data derived from human primary umbilical cord MSCs (UCMSCs) [12] and primary BMMSCs [56] as well as cultured endometrial MSCs [57]. By unsupervised clustering, six similar clusters were observed in cultured endometrial MSCs, BMMSCs, and WJMSCs (Figure S6A), including stem-like APCs (Cluster 1), MPCs (Cluster 2), preadipocytes (Cluster 3), pre-COs (Cluster 4), prechondrocytes (Cluster 5), and pre-SMCs (Cluster 6). This result suggested that cultured MSCs from different tissues shared similar subpopulation compositions. Interestingly, primary UCMSCs were mainly composed of Cluster 2 cells, while primary BMMSCs were mainly composed of Cluster 2 cells as well as cells from a novel BMMSC-specific cluster (Cluster 7) (Figure S6A). This primary BMMSC-specific cluster (Cluster 7) highly expressed hematopoietic stem cell (HSC)-niche factor genes, including *CXCL12* and *ANGPT1* (Figure S6B), and was thus referred to as the “HSC-niche support cluster”. The discrepancy in cluster groups between cultured and uncultured MSCs suggested that primary BMMSCs may lose their original gene expression activity related to HSC-niche support after *ex vivo* culturing, which is consistent with a previous finding that MSCs lose their HSC-niche function during culture [58]. In addition, the characteristic surface markers for MSCs, including *CD73/NT5E*, *CD90/THY1*, and *CD44*, were expressed higher in cultured MSCs than in primary MSCs both at the expression level and in terms of the percentage of positive cells (Figure S6C). This result suggested that these MSC characteristics might increase during culture. Taken together, our findings not only uncover the molecular and functional heterogeneity of cultured MSCs, but also pave a way for exploring the distinct heterogeneous characteristics between cultured and uncultured MSCs.

## Discussion

MSCs are considered as a promising candidate for cell-based regenerative medicine due to their self-renewal capacity, multilineage differentiation potential, paracrine effects, and immunosuppressive properties [59], [60]. However, whether and to what extent the MSC population contains heterogeneous subpopulations associated with diverse functions and characteristics remains largely unknown. In this study, we performed high-throughput scRNA-seq and a comprehensive analysis on *ex vivo*-expanded human BMMSCs and WJMSCs, which represent cell sources from adult and neonatal tissues, respectively. Our study identified the inherent cellular composition of MSCs, including a stem-like APC subpopulation, a multipotent progenitor subpopulation, a specific adipocyte precursor subpopulation, a specific osteo–chondrocyte precursor subpopulation, and an immunoregulatory prechondrocyte subpopulation, and provided a reconstruction of the transcriptional hierarchies of these subpopulations as well.

MSCs exhibit stemness characteristics *in vitro*, expanding rapidly and maintaining their morphology for up to 10 passages. However, there is still a lack of evidence to identify the stem cell populations among MSCs. In our data, Cluster 1 cells specifically expressed *CSPG4/MCAM/NES* and exhibited stemness signatures and negative regulation of differentiation pathways. Combining these properties with its location at the apex of the developmental trajectory, Cluster 1 was referred to as the stem-like APC cluster. Interestingly, MSCs were reported to be derived *in vivo* from NESTIN^bright^ NG2/CSPG4^+^ periarteriolar mesoderm progenitor cells, which possess the stemness characteristics of self-renewal through serial transplantations and multilineage mesodermal differentiation potential [21], [22], [23]. This suggests that stemness might be maintained in long-term culture as the Cluster 1 subpopulation. Moreover, stem cells in long-term culture show a strong proliferative ability to support successive rounds of replication and passaging without differentiation [61], [62], consistent with the highly proliferative phenotype of Cluster 1 cells. In addition, while Cluster 1 was closely related to NG2^+^ periarteriolar cells, Cluster 1 (APCs), Cluster 3 (preadipocytes), and Cluster 5 (prechondrocytes) have a comparatively higher similarity with LEPR^+^ perisinusoidal cells (Figure 2C). As LEPR^+^ cells serve as the major source of cartilage and adipocytes in adult mouse BM [63], the comparative similarity is supportive of the adipogenesis or chondrogenesis potentials of Clusters 1, 3, and 5.

Lineage priming is a molecular model of stem/progenitor cell (S/PC) differentiation in which S/PCs express low levels of a subset of genes associated with the differentiation pathways to which they can commit. Thus, they are “primed” for expression later during differentiation [64]. This concept has been widely used to explain the stochastic differentiation ability of HSCs. A similar process might occur with MSCs. Previous studies have shown that MSCs simultaneously express markers of more than one mesenchymal lineage [46], [64], suggesting the existence of a lineage-priming state in MSCs. However, the transcriptome pattern of lineage priming in MSCs and the relationship between lineage priming and lineage specification are incompletely understood. With the support of advanced scRNA-seq techniques, we revealed that MPCs, a subpopulation of MSCs, co-expressed lineage-associated TFs, markers, and receptors, suggesting the presence of a lineage-priming state specifically in MPCs. Furthermore, subclustering identified three lineage-biased subgroups, suggesting that lineage specification begins in multipotent progenitors with lineage priming. Together, our study extends the concept of lineage priming to MSCs and sheds light on the potential developmental continuum connecting stem cells to downstream precursors.

MSCs exhibit proinflammatory and anti-inflammatory properties [4], [65], [66]. However, whether these properties are exerted by homogeneous MSCs or by a distinct subset of cells remains elusive. Here, we found that the immunomodulatory function of MSCs was likely executed by a specific subpopulation (Cluster 5) instead of the entire MSC population. This subpopulation of cells specifically expressed genes related to proinflammatory and anti-inflammatory signatures, supporting the immunoregulatory plasticity of MSCs. We further identified specific surface markers for this cluster, including *CD106/VCAM1*, *CD47*, *CD248*, and *CD87/PLAUR*. CD106^+^ MSCs derived from placental chorionic villi have been demonstrated to be more effective in modulating T helper subsets [67], [68]. However, it is still unknown whether the immunoregulatory signatures are limited to CD106^+^ cells. Our study showed the specificity of this immunoregulatory subpopulation, which was identified as prechondrocytes and located at the end of the differentiation paths. Moreover, previous studies have shown that mature chondrocytes can exert an anti-inflammatory effect. For example, primary chondrocyte-derived exosomes can prevent osteoarthritis progression via expression of anti-inflammatory cytokines [69]. This research supports the findings of our study that prechondrocytes can express complex and varied immune signatures and likely produce exosomes or secrete factors that are the basis of the immunomodulatory function of MSCs. A recent study performing scRNA-seq on primary WJMSCs revealed distinct subpopulations defined by enrichment of terms related to proliferation, development, and inflammation response, but the specific markers and functional modulators for subpopulation identification need further investigation. Moreover, increasing evidence has shown that the immunoregulation functions exerted by MSCs are cell-contact dependent and/or produce various immunoregulatory and growth factors [4], [65], [66], [70]. As the specific immunomodulatory subpopulation (Cluster 5) was identified in our study, it is important to explore the pattern and the underlying mechanisms of its immunoregulatory effect in detail in the future.

MSCs from neonatal tissues show higher proliferative capacity than MSCs from adult tissues, and MSCs derived from BM significantly inhibit allogeneic T cell proliferation [19], [71]. By scRNA-seq analysis, we found that WJMSCs contained a higher percentage of proliferative stem-like APCs (Cluster 1) compared to BMMSCs (17.3% *vs*. 5%), supporting the biological superiority of WJMSCs in expansion [71], [72]. On the other hand, the superiority of BMMSCs in immunomodulation [72], [73] could be related to the dominance of the prechondrocyte subpopulation (Figure S2C, BMMSCs, 98.84% *vs*. WJMSCs, 1.16%) and the increased proportion of CD106^+^ cells (Figure S4E, BMMSCs, 72.73% ± 24.66 *vs*. WJMSCs, 23.78% ± 11.29). Therefore, the tissue-specific MSC characteristics can be potentially ascribed to the different proportions of functional clusters.

To our knowledge, scRNA-seq studies of different types of MSCs have been published, including the out-of-thaw MSCs, induced pluripotent stem cell (iPSC)-derived MSCs, and the *in vivo* primary MSCs. The potential phenotypic signatures of MSCs ware varying among these studies with some important differences in emphasis. For example, by comparing the pre-freeze and out-of-thaw samples, Medrano-Trochez et al. found that out-of-thaw MSCs exhibited higher levels of cholesterol/steroid biosynthesis and regulation of apoptosis, but lower levels of cytokine signaling, cell proliferation, and cell adhesion [74]. When investigating the gene regulatory networks during chondrogenesis from human iPSCs (hiPSCs), Wu et al. found that inhibiting Wnt signaling and melanocyte-inducing TF (MITF) could enhance the yield and homogeneity of hiPSC-derived chondrocytes [75]. A unique marrow adipogenic lineage precursor (MALP) was identified in primary BMMSCs that has been reproted to play critical roles in maintaining marrow vasculature and suppressing bone formation as an important part of niche cells [76]. Huang et al. profiled the single-cell transcriptomes of 361 UCMSCs from 7 samples under different conditions, revealing that human-derived MSCs had limited heterogeneity [9]. However, the cell number of each sample was relatively small (∼ 50 cells/sample) and they only analyzed the samples individually. The conclusion that MSCs had limited heterogeneity depended only on the similar 4 subclusters and gene expression patterns identified in each individual sample. Moreover, they also mentioned that the limited heterogeneity in these UCMSCs was strongly associated with the dominant cell cycle effect on MSCs. Comparatively, our study dissected the heterogeneity by integrated analysis of a large number of MSCs and intentionally removing the cell cycle effects, which would be more conducive for subpopulation identification. Additionally, cultured conditions might influence the expansion and differentiation ability of MSCs, which will also impact the heterogeneity of MSCs. Pattappa et al. reported that MSCs expanded with normoxia (20% oxygen) had more rapid initial proliferation but contained a greater proportion of senescent cells than those under hypoxia (5% or 2% oxygen). These phenomena were associated with the metabolic profiles from glycolysis [77]. Xie et al. also indicated that the metabolic profile of MSCs impacted their functional heterogeneity [78]. Thus, it should be noted that the effects of cellular metabolism should be considered during MSC culture and application.

In summary, we performed a comprehensive investigation of the heterogeneity of MSCs and discovered distinct subpopulations with specific characteristics, including stem-like APCs, multipotent progenitors, specific lineage precursors, and immunomodulatory prechondrocytes. We constructed the developmental hierarchies of cellular subpopulations among cultured MSCs for the first time. These transcriptional profiles identified MSC subsets and related them to specific markers that could be used to purify functional subpopulations for more specific and effective therapeutic applications.

## Materials and methods

### Isolation and culture of WJMSCs and BMMSCs

For isolation of WJMSCs and BMMSCs, fresh human umbilical cords and BM samples were obtained (Table S1). The umbilical cord was cut down into smaller segments. Then, the arteries and veins were removed and the remaining parts were immersed in a stem cell culture medium. BMMSCs were obtained by BM puncture aspiration of the iliac crest cavity from young children with cerebral palsy. Mononuclear cells were collected by Ficoll-based density gradient centrifugation and cultured in T75 flasks at a density of 160,000/cm^2^ in MEM alpha basic (Catalog No. C12571500BT MEM α, Nucleosides MEM, Invitrogen, Carlsbad, CA) culture medium supplemented with penicillin and streptomycin (Gibco, Carlsbad, CA) and 10% fetal bovine serum (Catalog No. IVGN-10099141, Gibco). Cells were cultured at 37 °C in a humidified atmosphere with 5% CO_2_. Cells were passaged and trypsinized with 0.25% trypsin/EDTA at 80%–90% confluence. MSCs intended for functional assays were harvested between passage 2 and passage 5. Cells from passage 6 or 7 were used for subsequent scRNA-seq analysis.

### Osteogenic lineage differentiation and staining analysis

MSCs were subcultured in 6-well plates at an initial density of 2 × 10^4^/cm^2^ with standard expansion medium. When the cells reached 60%–80% confluence, the medium was changed into 2 ml human osteogenic differentiation medium (Catalog No. HUXUC-90021, Cyagen Biosciences, China). The medium was refreshed every 3 days. After 2–4 weeks’ differentiation, cells were rinsed by Dulbecco’s Phosphate-Buffered Saline (DPBS; Catalog No. C14190500CP, Invitrogen) and fixed with 4% paraformaldehyde. Then the cells were stained with 1 ml Alizarin Red S solution for 3–5 min. Calcified matrix was stained red with Alizarin Red S, indicating the deposition of calcified matrixes on the osteogenic differentiated human MSCs.

### Adipogenic lineage differentiation and staining analysis

MSCs were subcultured in 6-well plates at an initial density of 2 × 10^4^/cm^2^ with standard expansion medium. When the cells were 100% confluent, the medium was changed into 2 ml osteogenic differentiation medium A (Catalog No. HUXUC-90031, Cyagen Biosciences). After 3 days, the medium was changed into 2 ml Adipogenic Differentiation Medium B. After alternating the A and B media 3–5 times (12–20 days), B medium was used for 4–7 days until a sizable amount of large and round lipid droplets emerged. During B medium maintenance culture, fresh B medium was replaced every 2–3 days. Then, cultured MSCs were rinsed by DPBS 1–2 times and were fixed for 30 min at room temperature with 4% paraformaldehyde. After the DPBS rinse, cells were stained with 1 ml Oil Red O solution for 30 min. Oil Red O imparts red–orange color to the lipid droplets.

### Chondrogenic lineage differentiation and staining analysis

MSCs for chondrogenic differentiation were cultured with chondrogenic differentiation medium (Catalog No. HUXUC-90041, Cyagen Biosciences) for 14 days. The chondrogenic aggregates were fixed with 10% formalin for 20 min and stained with Alcian Blue 8GX for 30 min.

### Flow cytometry

MSC samples were examined by flow cytometry analysis with the anti-human antibodies, including CD73 (CD73-PE; Catalog No. 12-0739-41, ThermoFisher Scientific, Waltham, MA), CD90 (CD90-PE; Catalog No. 12-0909-42, ThermoFisher Scientific), CD105 (CD105-APC; Catalog No. 17-1057-41, ThermoFisher Scientific), CD34 (CD34-APC; Catalog No. 17-0349-42, ThermoFisher Scientific), CD45 (CD45-PE; Catalog No. 12-0459-42, ThermoFisher Scientific), CD11b (CD11b-APC; Catalog No. 101212, BioLegend, San Diego, CA), CD19 (CD19-FITC; Catalog No. 555412, BD, Franklin Lake, NJ), and HLA-DR (HLA-DR-FITC; Catalog No. 11-9956-41, ThermoFisher Scientific). Cells were harvested and re-suspended in a staining buffer (2% fetal bovine serum in DPBS), and were subsequently incubated with corresponding antibodies at 4 °C for 30 min avoiding light. After the samples were washed with DPBS and re-suspended in staining buffer, they were run on a Moflo XDP (Bechman, CA). For each sample, more than 8000 events were acquired.

### T cell proliferation assay

Briefly, T cells were purified from peripheral blood samples by negative selection using the EasySep Human T cell Enrichment Kit (Catalog No. 17951, StemCell Technologies, Vancouver, Canada). Enriched T cells were stained with Cell Proliferation Dye eFluor 450 (Catalog No. 65-0842, ThermoFisher Scientific) to assess cell proliferation. Dye eFluor 450-labeled T cells (2.5 × 10^4^) were stimulated with anti-CD2/CD3/CD28 coated microbeads (Pan T Cell Activation Kit; Miltenyi Biotech, Bergisch Gladbach, Germany) or with uncoated microbeads as a negative control in a 1:10 bead:T cell ratio. These cells were cocultured with allogeneic CD106^+^ or CD106^−^ MSCs, which had been previously seeded in 96-well plates (5000 or 625 cells/well). The percentage of T cell proliferation was measured after 3.5 days in a Moflo XDP (Bechman, CA) and calculated by the percentage of FSC^high^Dye^low^ cells in the living cells gated by forward scatter (FSC) / side scatter (SSC). More than 8000 events were acquired for analysis. The data were normalized with respect to the percentage of activated T cells without coculture with MSCs.

### scRNA-seq library preparation and sequencing

Validated WJMSCs and BMMSCs were collected and re-suspended at 1 × 10^6^/ml in DPBS with 0.04% bovine serum albumin. Cells with higher aggregation rate (measured by a Countstar cell count and analysis system) were filtered to remove the cell aggregates. The cell suspensions (> 90% living cells examined by Countstar) were loaded on a Chromium Single Cell Controller (10x Genomics). The scRNA-seq libraries were sequenced on Illumina Hiseq X-ten platform with a 150-bp paired-end read length.

### scRNA-seq data processing

Raw sequencing data were processed by the Cell Ranger 3.0.2 pipeline with default parameters. Each sample was aligned to GRCh38 by the ‘Cell Ranger Count’ function to get the raw gene expression matrices. These matrices were further analyzed by Seurat (v3.0.2) for quality control and downstream analysis [79]. Low-quality cells that had less than 200 genes per cell and less than 3 cells per gene were discarded. Then to remove the outliers, cells were kept based on stringent criteria: 1000 < genes per cell < 6500, and percentage of mitochondrial genes < 0.05. After quality control, a total of 61,296 cells were retained.

### Dimension reduction, clustering, and identification of DEGs

The top 4000 most highly variable features that exhibit high cell-to-cell variation from each sample were selected for data integration. In our scRNA-seq experiments, six MSC samples were sequenced in three batches. Canonical correlation analysis was applied to remove the batch effect for data integration. Next, the top 20 principal components of the integrated data were selected for principal component analysis, UMAP analysis, and graph-based clustering (resolution = 0.15) to identify distinct subpopulations. DEGs were identified by the ‘FindAllMarkers’ function in Seurat (min.pct = 0.25, thresh.use = 0.25). Metascape [80] was used for pathway enrichment analysis.

### Pseudotime analysis

The Monocle2 package (v2.8.0) [81] was used to determine the pseudotime developmental relationships of each cluster in MSCs. We used top 3000 highly variable genes identified by Seurat to sort cells in pseudo-time order. The ‘DDRTree’ function was applied to reduce dimensions to infer the potential developmental path, and the ‘differentialGeneTest’ function was applied to identify DEGs along pseudo-time order. The remaining parameters were default.

### RNA velocity

RNA velocity was introduced to calculate the spliced and unspliced RNAs to indicate the transcriptional kinetic activity. A loom file with counts divided into spliced/unspliced/ambiguous of each gene in each cell was generated by velocyto.py on the BAM file from the CellRanger analysis. Only cells identical to the Seurat object (Cluster 1–Cluster 5) were retained for downstream analysis. Then RNA velocity was estimated by velocyto.R with default settings. The velocity fields were projected on to the UMAP embedding from the Seurat analysis.

### Integrated analysis of scRNA-seq datasets

Seurat (v3.0.2) was applied to integrate the public scRNA-seq datasets with our data. The top 4000 featured genes that were repeatedly variable across datasets were used to identify anchors across batches with the ‘FindIntegrationAnchors()’ function. The anchors were used to guide integration across multiple datasets with the ‘IntegrateData()’ function. The corrected data (integrated assay) were used for downstream analysis. We regressed out the difference between the G2/M and S phase scores with the ‘ScaleData’ function to remove cell cycle effects.

### TF regulon analysis

TF regulons were analyzed using SCENIC v1.1.2-2 workflow in R [82]. Normalized data from Seurat were used to generate the regulon activity score of TFs by default parameters. The average activity level of regulons in each cluster was calculated to show the main regulatory changes of different clusters through hierarchical clustering. Regulons showing significant difference in average activity between clusters were selected shown by heatmap.

### Data comparison

The publicly available dataset of LEPR^+^ and NG2^+^ cells was downloaded from Gene Expression Omnibus (GEO: GSE128423; https://www.ncbi.nlm.nih.gov/geo/query/acc.cgi?acc=GSE128423). The publicly available dataset of multiple mesoderm lineages was downloaded from Sequence Read Archive (SRA: PRJNA319573; https://www.ncbi.nlm.nih.gov/bioproject/PRJNA319573/). The publicly available dataset of osteoblasts and chondrocytes was downloaded from GEO (GEO: GSE106292; https://www.ncbi.nlm.nih.gov/geo/query/acc.cgi?acc=GSE106292). The publicly available dataset of BMMSCs was downloaded from GEO (GEO: GSE147287; https://www.ncbi.nlm.nih.gov/geo/query/acc.cgi?acc=GSE147287). The publicly available dataset of cultured endometrial MSCs was downloaded from GEO (GEO: GSE149651; https://www.ncbi.nlm.nih.gov/geo/query/acc.cgi?acc=GSE149651). The publicly available dataset of primary UCMSCs was downloaded from SRA (SRA: PRJNA643879; https://www.ncbi.nlm.nih.gov/bioproject/?term=PRJNA643879). The same single-cell analysis approach in this study was applied to public scRNA-seq data. Then we quantified the correlation of single-cell clusters based on average gene expression of the typical gene signatures related to specific characteristics. Moreover, we performed Combat to remove the batch effects [83].

### Statistical analysis

Statistical analysis of the data was performed by a one-way analysis of variance (ANOVA) followed by Tukey’s post-hoc test among those with more than two groups. Statistical analysis of the data was performed by *t*-test between two groups. *P* < 0.05 was considered statistically significant. Analyses were performed using R packages or GraphPad Prism 8.

## Ethical statement

Ethical approval for the protocol of this open labeled, self-controlled trial was granted by the Ethics Committee of the General Hospital of the Chinese People’s Armed Police Forces (No. 200804). Written informed consent was obtained from every healthy donor, and the Ethics Committee of 3rd Medical Center, General Hospital of Chinese PLA approved the protocols.

## Data availability

Raw data of scRNA-seq have been deposited in the Genome Sequence Archive [84] at the National Genomics Data Center, Beijing Institute of Genomics, Chinese Academy of Sciences / China National Center for Bioinformation (GSA: HRA000220), and are publicly accessible at https://ngdc.cncb.ac.cn/gsa-human. The expression matrix reported in this study has been deposited in the OMIX, Beijing Institute of Genomics, Chinese Academy of Sciences / China National Center for Bioinformation (OMIX: OMIX745), and are publicly accessible at https://ngdc.cncb.ac.cn/omix. All other data supporting the findings of this study are available from the corresponding authors on reasonable request.

## CRediT author statement

**Chen Zhang:** Investigation, Data curation, Formal analysis, Validation, Visualization, Writing - original draft, Writing - review & editing. **Xueshuai Han:** Investigation, Data curation, Formal analysis, Visualization, Writing - original draft, Writing - review & editing. **Jingkun Liu:** Validation, Investigation. **Lei Chen:** Validation. **Ying Lei:** Validation. **Kunying Chen:** Validation. **Jia Si:** Formal analysis. **Tian-yi Wang:** Writing - review & editing. **Hui Zhou:** Resources. **Xiaoyun Zhao:** Resources. **Xiaohui Zhang:** Resources. **Yihua An:** Resources. **Yueying Li:** Conceptualization, Writing - original draft, Writing - review & editing, Project administration, Funding acquisition. **Qian-Fei Wang:** Conceptualization, Writing - review & editing, Supervision, Funding acquisition. All authors have read and approved the final manuscript.

## Competing interests

The authors have declared no competing interests.
