# Supplementary material for: Single-cell Transcriptomic Analysis Reveals the Cellular Heterogeneity of Mesenchymal Stem Cells
Source: Genomics Proteomics Bioinformatics. 2022 Feb 3;20(1):70–86. doi: 10.1016/j.gpb.2022.01.005 (PMC9510874; doi:10.1016/j.gpb.2022.01.005)
Supplement: Supplementary Table S1 [file mmc1.docx]

|  | **BMMSC1** | **BMMSC2** | **BMMSC3** | **WJMSC1** | **WJMSC2** | **WJMSC3** |
| --- | --- | --- | --- | --- | --- | --- |
| Age | 2-year-old | 2-year-old | 2.5-year-old | Unknown | Unknown | 29-year-old |
| Medical condition | Cerebral palsy | Cerebral palsy | Cerebral palsy | Eutocia, no genetic disorders | Eutocia, no genetic disorders | Eutocia, no genetic disorders |
| Sex | Female | Male | Female | Female | Female | Female |
| Passage | 6 | 6 | 7 | 6 | 6 | 6 |
| Cell viability | 89.89% | 90.11% | 81.99% | > 90% | 95.73% | 92.99% |
| Aggregation rate | 10.39% | 19.23% | 12.73% | ≈ 20% | 38.92% | 8.36% |
| **Sequencing information** | | | | | | |
| Cell number | 13,360 | 14,071 | 12,617 | 12,756 | 12,553 | 11,154 |
| Mean read count per cell | 56,759 | 52,158 | 58,706 | 66,324 | 75,602 | 68,228 |
| Median gene count per cell | 2896 | 2202 | 2937 | 4133 | 3838 | 4317 |
| Median UMI count per cell | 11,325 | 7263 | 11,667 | 25,658 | 19,129 | 19,652 |
| Total genes detected | 21,613 | 21,212 | 20,969 | 22,864 | 22,319 | 22,139 |
| Sequencing saturation | 60.80% | 59.80% | 57.80% | 40.40% | 56.60% | 51.30% |
| Number of remaining cells after filtering | 10,075 | 7499 | 10,128 | 11,655 | 11,872 | 10,067 |

**Table S1 Sample Information.**

*Note*: UMI, unique molecular identifier; WJMSC, Wharton's jelly-derived mesenchymal stem cell; BMMSC, bone marrow-derived mesenchymal stem cell.
